# Chondroid Syringoma of the Inner Corner of the EyeCase Report

**DOI:** 10.3390/reports9020136

**Published:** 2026-04-28

**Authors:** Alin Tatu, Tiberiu Tebeica, Mihaela Denisa Pirvu, Cristian Constantin Popa, Valeriu Ardeleanu

**Affiliations:** 1 Faculty of Medicine, “Lower Danube” University, 800008 Galati, Romania; 2 “St. Parascheva” Infectious Diseases Hospital, 700116 Galati, Romania; 3 Leventer Medical Center, 011864 Bucharest, Romania; 4 First Surgery Department, “St. Andrew” Emergency County Clinical Hospital, 900591 Constanta, Romania; 5 Department of General Surgery, Faculty of Medicine, “Ovidius” University, 900470 Constanta, Romania; 6 Second Department of General Surgery, University Emergency Hospital, 050098 Bucharest, Romania; 7 Department of General Surgery, “Carol Davila” University of Medicine and Pharmacy, 050474 Bucharest, Romania; 8 Department of General Surgery, Plastic Surgery, Faculty of Medicine, “Ovidius” University, 900470 Constanta, Romania; 9 “CFR” General Hospital, 800225 Galati, Romania; 10 Arestetic Clinic, 85 Brailei Street, 800306 Galati, Romania

**Keywords:** chondroid syringoma, histological, immunohistochemistry, molecular, markers, surgery, laser, electrofrequency, dermoscopy, case report

## Abstract

**Background and Clinical Significance:** Chondroid syringoma is a very rare tumor arising from the sweat glands, with an incidence described in the literature of 0.01% of all primary skin tumors. **Case presentation:** This paper aims to present the case of a patient treated in our clinic for a large cyst located at the inner corner of the left eye, which appeared two years ago and progressively increased in size. The patient presented for cosmetic reasons and discomfort, especially when wearing glasses. The diagnosis of chondroid syringoma is generally established clinically. The differential diagnosis includes other benign cutaneous lesions (pleomorphic adenoma, lipoma, neurofibroma, a dermoid cyst, dermatofibroma, pleomorphic adenoma of the salivary glands, a sebaceous cyst, or hemangioma) or malignant lesions (basal cell carcinoma, squamous cell carcinoma, or adenocarcinoma). Additional imaging investigations—CT and MRI—are rarely required and would mainly assess the extent of the lesion. Dermoscopy is an early differential diagnostic method, especially for small lesions of 1–3 mm, such as xanthelasma, milia, or basal cell carcinoma. Chondroid syringoma may be treated using minimally invasive methods such as fractional CO_2_ laser, radiofrequency, or electrocautery, but only when the lesion is superficial and small. For larger and deeper tumors, such as in our case, multiple treatment sessions would be required, increasing the cost, and complete removal would not be guaranteed. **Conclusions:** The chosen treatment is surgical excision with oncologic margins, followed by histopathological and immunohistochemical examination to prevent recurrence and assess the risk of malignancy.

## 1. Introduction and Clinical Significance

Chondroid syringoma, also known as a mixed tumor of the skin, is a rare cutaneous lesion that is benign in most cases and is characterized by the simultaneous presence of epithelial, myoepithelial, and mesenchymal components within the same tumor. Its incidence is 0.01% of all primary skin tumors [1,2].

The most common location of chondroid syringembryonicoma is in the head and neck region, while only a minority of cases occur in the periorbital area [3,4]. In all of the medical literature, only a few dozen cases have been reported on the eyelids, nose, and paranasal sinuses and lips [5,6,7,8,9]. Additionally, lesions have been described on the trunk (including the lumbar region and axilla) as well as on the fingers [10].

Other studies also report its rare occurrence on the trunk, where it has been misdiagnosed as a lipoma [11].

There is a male predominance, and the average age of patients diagnosed with chondroid syringoma ranges between 20 and 60 years [12].

The clinical manifestations of chondroid syringoma are nonspecific. It typically presents as a painless subcutaneous nodule and is often misdiagnosed as an epidermoid cyst or lipoma [11]. The risk of cellular atypia and malignancy is the reason why surgical treatment should not be delayed. Here, we report the case of a patient who required complete excision of a chondroid syringoma located at the inner corner of the eye. We considered the clinical presentation, histological findings and treatment, along with a review of the relevant literature.

## 2. Case Presentation

We report the case of a 62-year-old man admitted to our clinic for a large cystic lesion located at the inner corner of the left eye. The patient requested surgical intervention for aesthetic reasons and due to the discomfort caused by wearing glasses.

Methods were based on anamnesis, evaluation of personal pathological antecedents, history of the condition, clinical diagnosis, specialized treatment, confirmation of the diagnosis through anatomopathological and immunohistochemical tests and, last but not least, the six-month postoperative follow-up.

The lesion had appeared approximately two years earlier and had shown progressive growth in size.

His medical, family, and psychosocial history revealed no significant findings, and the patient was unaware of any genetic predisposition.

**Figure 1:**
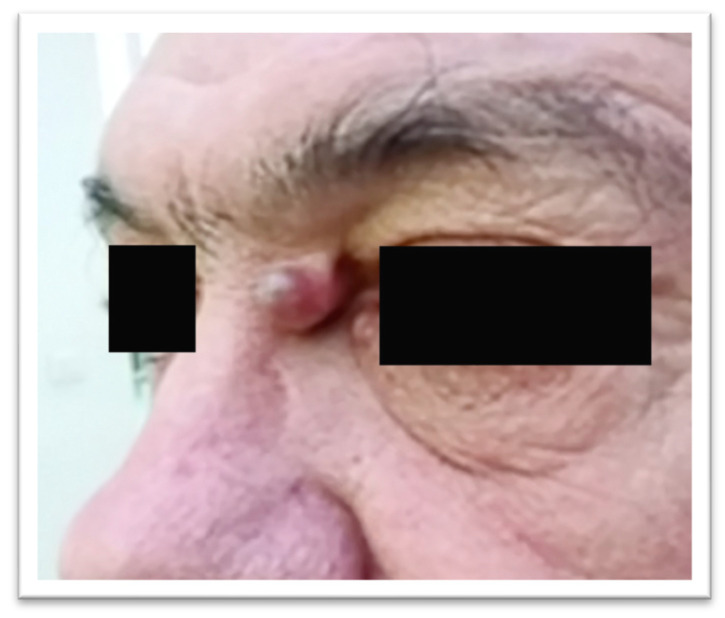
Tumor of the medial canthal area.

Clinical examination revealed a round lesion of approximately 1 cm, with a firm-elastic consistency, relatively mobile over the deeper planes, painless, and located about 5 mm from the medial canthus of the left eye (Figure 1).

The mass was excised under local anesthesia, and reconstruction was performed using local advancement flaps.

The specimen was sent for histopathological examination.

**Figure 2:**
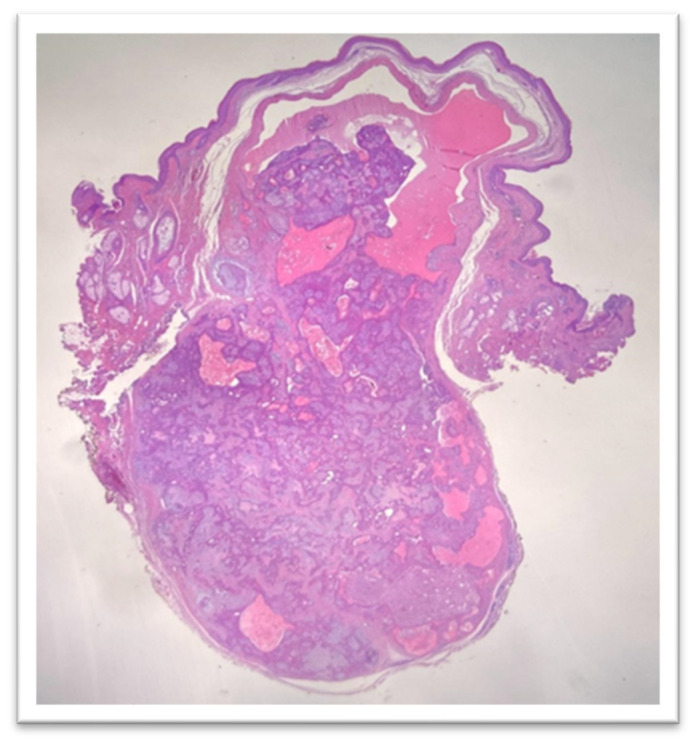
Histological examinationHE, 12.5×: ductal differentiation arranged in a stroma with a mixed fibrous, myxoid and fibroid appearance.

**Figure 3:**
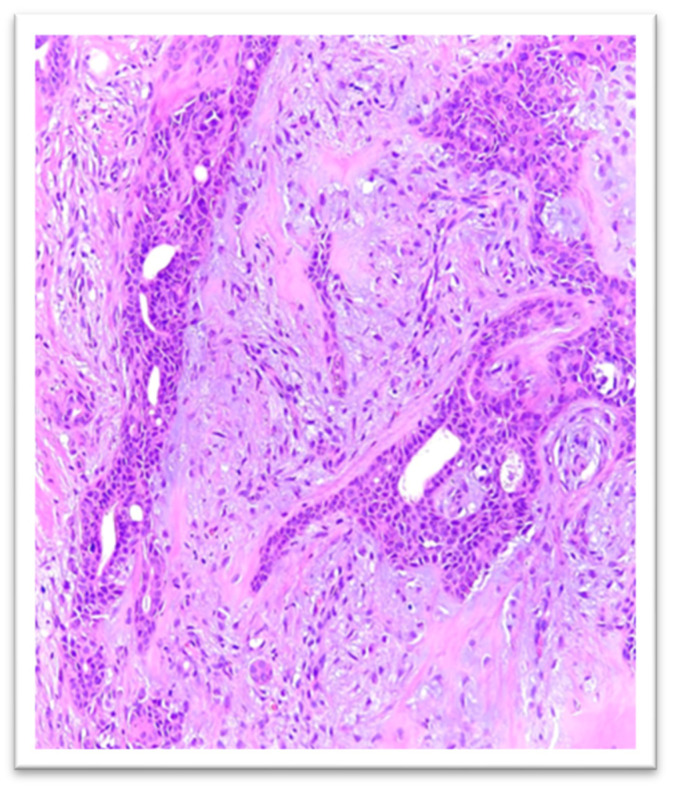
Histological examination—HE, 200×: flattened epidermis on the surface of a dermal nodule—composed of trabecular plaques and cords of epithelial structure.

Histologic examination showed a flattened epidermis overlying a well-circumscribed dermal nodule composed of intersecting nests and cords of epithelial structures with ductal differentiation, arranged within a stroma exhibiting mixed fibrous, myxoid, and fibroid features (Figure 2 and Figure 3).

**Figure 4:**
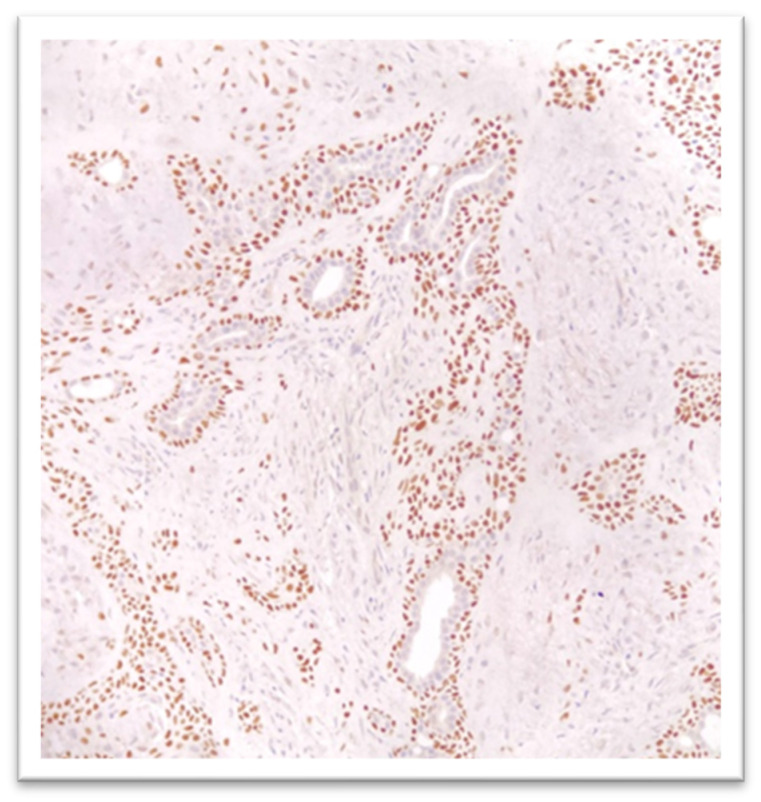
Immunochemistry—the presence of the p63 marker in a population of tumor cells: p63 was positive and CD56 and synaptophysin were negative (40×).

Immunohistochemistry showed p63 positivity in a population of tumor cells—mostly of myoepithelial origin—located at the periphery of the epithelial structures, while CD56 (NCAM) and synaptophysin were negative (Figure 4). Following histologic evaluation, the diagnosis of chondroid syringoma was established, with no evidence of cellular atypia.

At the 6-month postoperative follow-up, no local recurrence was observed, and the scar showed a favorable aesthetic outcome.

## 3. Discussion

Chondroid syringoma was first described in 1859 by Billroth as a tumor resembling a benign mixed tumor of the salivary glands. Later, Hirsch and Helwig identified the presence of sweat glands within a sweat stroma and proposed five histopathological criteria for diagnosing this lesion. For an accurate diagnosis, chondroid syringoma may fulfill all five histopathological criteria, but it can also be confirmed when only a single distinctive feature is present [13].

The tumor originates from apocrine or eccrine sweat glands, but it may also result from idiopathic proliferation of an ectopic embryonic rest. Some studies suggest that local trauma may represent a predisposing factor for the development of chondroid syringoma [14]. In our case, the patient did not report any trauma in the region; however, wearing glasses could represent a source of chronic local compression.

The size of chondroid syringoma usually ranges between 0.5 and 3 cm, presenting as a slow-growing and painless mass [15]. In our case, the tumor measured 1 cm and had been present for approximately two years before presentation.

Clinically, chondroid syringoma may be mistaken for neurofibroma, a dermoid cyst, a sebaceous cyst, dermatofibroma, pleomorphic adenoma of the salivary glands, or even squamous cell carcinoma or basal cell carcinoma [6]. Another important differential diagnosis is lipoma, as numerous cases of chondroid syringoma located on the back have been initially interpreted as lipomatous tumors [11]. In our case, the initial clinical diagnosis was a sebaceous cyst or hemangioma, considering the bluish coloration of the lesion.

Although most reported cases occur in patients between 20 and 60 years of age, the authors of one study described a case of chondroid syringoma located on the cheek of a 7-year-old child [16]. Although slow growth is characteristic of this type of tumor, in that case, the lesion tripled in size within only four months. Such reports highlight the importance of including chondroid syringoma in the differential diagnosis of tumors with suggestive clinical features, regardless of the patient’s age.

There are certain anatomical particularities at the level of the internal angle of the eye. The medial canthal area represents a delicate anatomical region where the fields of ENT, ophthalmology, plastic surgery, and general surgery intersect. It contains fine structures with essential functional roles and often requires multidisciplinary teams for oncologic resections or complex reconstructions [17].

The medial canthus is the inner corner of the eye, toward the nose, representing one of the most delicate regions of the face. It is the point where the upper and lower eyelids meet and contains a complex anatomical structure that includes ligaments, muscles, and lacrimal components—the superior lacrimal canaliculus, the inferior lacrimal canaliculus, and the lacrimal sac (in close proximity), as well as the nasolacrimal duct and the medial canthal tendon. It is an essential area for lacrimal drainage and for maintaining eyelid stability.

The muscular, ligamentous, vascular, and neural structures of this region have particular anatomical and surgical importance, as they determine both the operative access and the risk of functional and aesthetic complications.

The muscular structures of anatomical and surgical importance in this region include: muscles of the upper eyelid (the orbicularis oculi muscle, fibers of the levator palpebrae superioris, the superior tarsal muscle—Müller), retractors of the lower eyelid (the inferior rectus muscle; accessory fibers arising from the inferior rectus that form the capsulopalpebral fascia of the medial rectus, including the medial check ligament, which stabilizes eyelid movement; and the inferior tarsal muscle).

**Figure 5:**
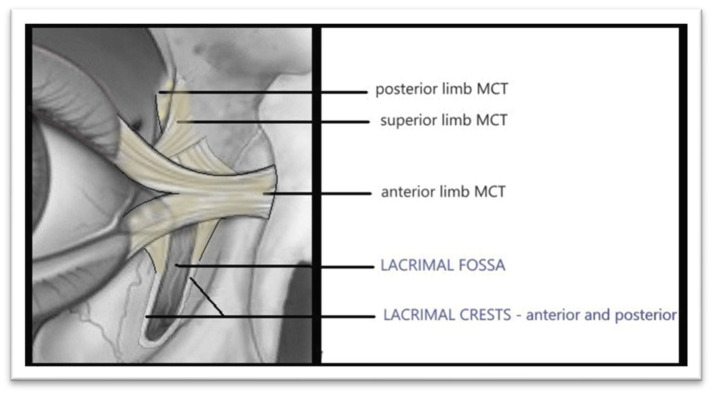
Ligamentous structuresMedial Canthal Tendon (drawn by the authors).

The ligamentous structures include: the medial canthal tendon, the posterior lacrimal ligament and the anterior lacrimal ligament (Figure 5).

Regarding the vascular supply, it should be noted that the arterial blood flow originates from branches of the angular artery (the terminal branch of the facial artery) and branches of the dorsal nasal artery (from the ophthalmic artery). These form a rich arterial network that bleeds abundantly during surgical procedures. The venous drainage of the region is directed predominantly toward the angular and nasal veins, with connections to the periorbital venous plexus [18,19].

**Figure 6:**
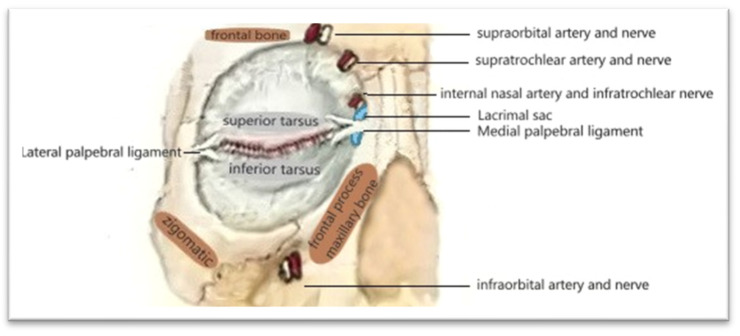
Blood supply and innervation of the periorbital region—drawn by the authors.

The sensory innervation of the region is provided by branches of the infratrochlear and supratrochlear nerves, as well as terminal branches of the infraorbital nerve, which supply sensation to the medial canthus and upper nasal area. Motor innervation derives from facial nerve (VII) fibers that activate the orbicularis oculi muscle [20] (Figure 6).

In the context of tumor resections in this region, injuries to the medial canthal tendon may occur. Such injuries can lead to avulsion of the posterior branch of the tendon, which weakens this structure and subsequently results in lateral traction, causing eyelid deformities and impaired eyelid function [21].

The diagnosis of chondroid syringoma is established through both clinical and paraclinical evaluation.

Clinically, chondroid syringoma typically presents as a solitary, well-defined nodule with a firm or elastic consistency and very slow growth, usually over the course of several years. The lesion is generally asymptomatic, with a normal skin color or a slightly yellowish/pink hue, without ulceration and without inflammatory signs [22].

The dermoscopic appearance is rarely described in the literature. Some studies reveal the nodular nature of the lesion, usually with well-defined borders characteristic of benign tumors. The pattern is typically described as yellowish homogeneous areas corresponding histologically to the cystic components and the myxoid–chondroid stroma, as well as translucent areas with a “gelatinous/clear” appearance (sometimes referred to as hypopigmented “lakes”); they reflect the mucin-rich stroma, and are sometimes blue-gray when the stroma is dense. The lesion can also exhibit the presence of blood vessels, subtle vascular structures, usually fine, linear or very delicate arborizing vessels that are much less prominent than in basal cell carcinoma. Occasionally, scattered dotted vessels may be observed. The absence of pigment structures typical of melanocytic lesions has been noted [23].

It is important to note that, given the rarity of this tumor and its deep location, definitive diagnosis relies primarily on complete excision followed by histopathological and immunohistochemical examination [24,25].

Fine-needle aspiration (FNA) may be considered in the evaluation of chondroid syringoma, as it is a commonly used procedure for assessing nodules or suspicious masses. However, its accuracy is limited in this tumor, because the mixed epithelial–myoepithelial architecture and stromal components may not be adequately captured through aspiration [26].

A differential diagnosis of chondroid syringoma can be made with benign tumors such as: apocrine or eccrine hidrocystoma, cutaneous pleomorphic adenoma, dermoid or epidermoid cysts, pilomatricoma, chondroma, and benign neural tumors such as neurofibroma or schwannoma [1,25]. Chondroid syringoma and hidrocystoma share common periocular presentations, but hidrocystoma lacks the characteristic chondromyxoid stroma of chondroid syringoma. Cutaneous pleomorphic adenoma shows morphological similarities, including epithelial and myoepithelial components, but typically arises from salivary gland tissue. Dermoid and epidermoid cysts are cystic rather than solid lesions, whereas pilomatricoma displays shadow cells and calcifications. Neural tumors are S100-positive and lack ductal differentiation [27].

Also to be considered are malignant tumors, particularly basal cell carcinoma, adnexal carcinomas, malignant myoepithelioma, and carcinoma ex pleomorphic adenoma. Basal cell carcinoma is the most common clinical presentation in the periocular region, but histologically it shows peripheral palisading and stromal retraction—features that are absent in chondroid syringoma [14]. Malignant adnexal tumors exhibit marked atypia, necrosis, and infiltrative growth, whereas chondroid syringoma is well circumscribed and shows low proliferative activity. Carcinoma ex pleomorphic adenoma shares morphological and molecular features, including possible PLAG1 rearrangements, but demonstrates clear malignant epithelial transformation [28].

Definitive diagnosis is based on a combination of histopathology, immunohistochemistry, and, when possible, molecular analysis.

Regarding the histopathological examination, on hematoxylin–eosin staining, chondroid syringoma appears as an epithelial, myoepithelial, and mesenchymal neoplasm with a multiphenotypic character. Published studies to date have highlighted its distinctive morphology, with the tumor stroma described as fibrous, myxoid, chondroid, lipogenic, or even osteogenic [26]. Osseous formation is rare, with only six cases reported in the literature [27].

For an accurate diagnosis, immunohistochemistry is useful, highlighting the dual epithelial and mesenchymal differentiation characteristic of chondroid syringoma. A specific panel of markers is used in the evaluation of this tumor, including SOX10, p63, S100, calponin, SMA, EMA, c-KIT, CEA, and various cytokeratins. SOX10 is a sensitive marker for pleomorphic adenoma; although it is not specific to it, P63, a protein encoded by a gene located on chromosome 3q27–29, is expressed in the basal cells of epithelia and plays an essential role in the development and maintenance of epithelial structures [29].

In our case, histologic examination revealed the presence of epithelial nests and cords at the surface of the nodule, showing ductal differentiation. These structures were arranged within a mixed stroma composed of fibrous and myxoid components, a pattern suggestive of a tumor of sweat gland origin. For an accurate diagnosis, we performed immunohistochemical analysis. The p63 marker was positive, while CD56 and synaptophysin were negative, these markers being used primarily for differential diagnosis. CD56 is a marker specific to natural killer (NK) cells and plays an important role in host defense against tumors [30].

A genuine concern regarding the patient was the possibility that the lesion might be a malignant tumor. Benign chondroid syringoma generally does not show a tendency toward malignant transformation. However, extremely rare cases of malignant chondroid syringoma (cutaneous mixed carcinoma) have been reported in the literature [31,32,33]. Most of these cases appear to arise de novo. There are no extensive statistics regarding the rate of malignant transformation, given the rarity of both entities: the benign form is very uncommon, and the malignant form is even rarer, accounting for approximately 0.01% of cutaneous tumors [32].

Chronic inflammation, local microtrauma, and certain genetic alterations—such as PLAG1 gene rearrangement—have been suggested as possible factors involved in the development of malignant forms. The malignant tumor typically presents as a larger mass than the benign variant—often exceeding 3 cm—and may be accompanies by ulceration or pain [24,31].

In malignant forms, dermoscopy may reveal more atypical and disorganized features, with poorly defined borders. Irregular blue-gray areas and heterogeneous whitish structures may be present, reflecting invasive growth and stromal disruption. Pigment-like structures or areas of asymmetry may also appear, although they are nonspecific and often ill-defined. Vascular patterns tend to be more prominent and atypical, with irregular linear, polymorphous, or arborizing vessels, indicating increased angiogenesis and invasive behavior [34].

Malignant tumors may metastasize both lymphatically and hematogenously, involving lymph nodes, bone, or lungs. Assessment of local and distant extension may require imaging studies such as MRI and CT [35].

Histologically, malignant forms of chondroid syringoma show cytologic atypia, infiltrative margins, satellite nodules, and tumor necrosis, with frequent invasion of deeper tissues. Studies have shown that these malignant variants generally do not arise from a pre-existing benign chondroid syringoma but instead occur de novo. The most common location is the trunk, and they may occasionally be associated with other cutaneous lesions of the thoracic region [36,37].

P63 is a protein from the p53 family, frequently used as a marker in the diagnosis of cutaneous tumors [38]. It plays an important role in the differentiation and proliferation of epithelial cells and in identifying myoepithelial and epithelial cells, particularly within the sweat glands [39]. This marker contributes to the positive diagnosis of benign syringoma and helps differentiate it from other cutaneous tumors, such as basal cell carcinoma, which exhibits a different p63 expression pattern [24].

P63 may also be positive in the malignant form of chondroid syringoma; however, its expression varies according to the degree of tumor differentiation. In undifferentiated forms, its expression is often weaker or uneven, suggesting a loss of myoepithelial characteristics [40].

The immunohistochemical marker p16 is used to assess the degree of differentiation and estimate malignant potential, as it is involved in cell-cycle regulation as an inhibitor of cyclin-dependent kinases [33,41]. In benign chondroid syringoma, p16 is typically negative. The association of p16 negativity with p63 positivity may support the benign nature of the lesion. Positivity for p16 may suggest a more aggressive tumor behavior.

P16 is frequently used in the diagnosis of malignant basal cell carcinoma; however, it is not a specific marker and must be interpreted within the context of a broader immunohistochemical panel, which may include p63, Ki67, CK5/6, and EMA. This set of markers is useful in differentiating cutaneous tumors such as basal cell carcinoma, malignant pleomorphic adenoma, and chondroid syringoma [42].

Malignant pleomorphic adenoma shares similarities with malignant chondroid syringoma; however, differential diagnosis can be achieved through analysis of the genetic profile, particularly by identifying its location and specific genetic rearrangements such as those involving the PLAG1 gene (pleomorphic adenoma gene 1). This gene, located on chromosome 8q12, functions as a proto-oncogene and encodes a transcription factor involved in regulating gene expression and controlling cellular development. PLAG1 rearrangements may lead to the development of benign or, more rarely, malignant tumors [43]. Because of its role in regulating the development of myoepithelial and epithelial cells, PLAG1 is frequently implicated in tumors originating from the salivary and sweat glands. This genetic feature represents an important element in distinguishing malignant pleomorphic adenoma from malignant chondroid syringoma—two histologically similar entities but with different clinical implications and prognostic outcomes.

This molecular marker, assessed through FISH techniques, can contribute to the differential diagnosis of tumors with a mixed component. In benign forms of chondroid syringoma, genetic rearrangements such as those involving the PLAG1 gene may be present [44]. However, malignancy typically involves additional genetic alterations. Markers such as Ki67 or p16 may be used to evaluate the degree of tumor aggressiveness.

Microscopically, malignant forms show marked atypia, frequent mitoses, tumor necrosis, and local invasion. These tumors may be p16-positive, display a more disorganized architectural pattern, and exhibit a chondromyxoid stroma composed of epithelial and mesenchymal components [28,36,41].

The treatment of choice for chondroid syringoma is complete surgical excision, adapted to the anatomical particularities of the region. A proper assessment of the globe and periocular structures is essential for successful surgery. The primary objective is to preserve normal eyelid function, ensure adequate support of the lacrimal drainage system, and protect the globe [45].

The principles, technique, and strategy of the surgical treatment include protecting the anatomical structures of the region and making a small incision oriented along the natural skin lines (oblique or vertical within the medial canthal fold, or parallel to the eyelid margin). Given the narrow operative field, magnified dissection (using loupes), meticulous hemostasis, and fine, atraumatic instruments are preferred. The dissection is performed layer by layer (epidermis, dermis, subcutaneous tissue, and, if present, fibrous planes), followed by complete en bloc excision of the lesion with minimal but safe margins, avoiding unnecessary sacrifice of functional structures. Hemostasis must be delicate, achieved through compression or fine bipolar coagulation to prevent necrosis and retractile scarring, and closure is performed without tension, using fine sutures aligned with the natural skin lines, in one or two layers.

Reconstruction of medial canthal defects can be challenging. In certain cases, the use of multiple local flaps can cover tissue defects with minimal tension, provide a favorable cosmetic result, and ensure effective structural reconstruction [46].

Chondroid syringoma can be treated using minimally invasive methods such as fractional CO_2_ laser, radiofrequency, or electrocautery. CO_2_ laser treatment works by vaporizing the target tissue: the concentrated light beam is absorbed by the water within the lesion’s cells, causing them to heat and vaporize layer by layer. Radiofrequency destroys pathological tissue through heat generated by a high-frequency electric current, producing thermal coagulation of the lesion, which subsequently detaches and decreases in size over several days. Electrocautery may be used for controlled burning of lesions [47,48].

Among the advantages of these methods are superior cosmetic outcomes, minimal scarring, the possibility of treating multiple lesions simultaneously, reduced post-procedural recovery time, and a low risk of complications.

However, these methods are particularly effective for superficial and small lesions, contributing to their destruction and to skin smoothing. In the case of larger and deeper tumors, such as in our situation, multiple treatment sessions would be required, which would increase costs, and complete removal would not be guaranteed [49].

## 4. Conclusions

Chondroid syringoma is an extremely rare skin lesion, and is benign in most cases. Located most frequently in the head and neck region, it has a complex, mixed component.

Clinical manifestations are nonspecific, usually presenting as a painless subcutaneous nodule.

Differential diagnosis includes multiple other benign or malignant skin lesions.

Dermoscopy may serve as an early adjunctive tool in differential diagnosis, particularly when evaluating small, skin-colored papules.

The treatment of choice, especially in the case of large formations, is surgical excision within oncological limits. In this way, the formation is removed, the risk of cellular atypia and malignancy is assessed, and recurrence is prevented. Subsequently, while histopathological examination establishes a positive diagnosis, differential diagnosis problems may arise. Immunohistochemistry confirms the diagnosis, followed by the introduction of specific therapeutic management for this rare skin lesion.

## Data Availability

The original data presented in the study are included in the article, further inquiries can be directed to the corresponding author.

## Abbreviations

The following abbreviations are used in this manuscript:

SMA	Smooth Muscle Actin
NK-cell	Natural killer cell
EMA	Epithelial Membrane Antigen
c-KIT	Receptor tyrosine kinase
PLAG1	Pleomorphic Adenoma Gene 1
FISH	Fluorescence In Situ Hybridization
CEA	Carcinoembryonic Antigen
NCAM	Neural Cell Adhesion Molecule
